# Impact of an Automated Population-Level Cirrhosis Screening Program Using Common Pathology Tests on Rates of Cirrhosis Diagnosis and Linkage to Specialist Care (CAPRISE): Protocol for a Pilot Prospective Single-Arm Intervention Study

**DOI:** 10.2196/56607

**Published:** 2024-05-22

**Authors:** Joan Ericka Flores, Christina Trambas, Natasha Jovanovic, Alexander J Thompson, Jessica Howell

**Affiliations:** 1 Department of Gastroenterology St Vincent's Hospital Melbourne Fitzroy Australia; 2 Department of Medicine University of Melbourne Parkville Australia; 3 Department of Pathology St Vincent's Hospital Melbourne Aruba; 4 Disease Elimination Program Burnet Institute Melbourne Australia; 5 Department of Epidemiology and Preventive Medicine Monash University Clayton Australia

**Keywords:** noninvasive tests, cirrhosis, population health, screening, liver cirrhosis, liver, compensated, risk factor, pathology, population based, liver screening, prevalence, hepatocellular carcinoma, transient elastography, FibroScan

## Abstract

**Background:**

People with compensated cirrhosis receive the greatest benefit from risk factor modification and prevention programs to reduce liver decompensation and improve early liver cancer detection. Blood-based liver fibrosis algorithms such as the Aspartate Transaminase–to-Platelet Ratio Index (APRI) and Fibrosis-4 (FIB-4) index are calculated using routinely ordered blood tests and are effective screening tests to exclude cirrhosis in people with chronic liver disease, triaging the need for further investigations to confirm cirrhosis and linkage to specialist care.

**Objective:**

This pilot study aims to evaluate the impact of a population screening program for liver cirrhosis (CAPRISE [Cirrhosis Automated APRI and FIB-4 Screening Evaluation]), which uses automated APRI and FIB-4 calculation and reporting on routinely ordered blood tests, on monthly rates of referral for transient elastography, cirrhosis diagnosis, and linkage to specialist care.

**Methods:**

We have partnered with a large pathology service in Victoria, Australia, to pilot a population-level liver cirrhosis screening package, which comprises (1) automated calculation and reporting of APRI and FIB-4 on routinely ordered blood tests; (2) provision of brief information about liver cirrhosis; and (3) a web link for transient elastography referral. APRI and FIB-4 will be prospectively calculated on all community-ordered pathology results in adults attending a single pathology service. This single-center, prospective, single-arm, pre-post study will compare the monthly rates of transient elastography (FibroScan) referral, liver cirrhosis diagnosis, and the proportion linked to specialist care in the 6 months after intervention to the 6 months prior to the intervention.

**Results:**

As of January 2024, in the preintervention phase of this study, a total of 120,972 tests were performed by the laboratory. Of these tests, 78,947 (65.3%) tests were excluded, with the remaining 42,025 (34.7%) tests on 37,872 individuals meeting inclusion criteria with APRI and FIB-4 being able to be calculated. Of these 42,025 tests, 1.3% (n=531) had elevated APRI>1 occurring in 446 individuals, and 2.3% (n=985) had elevated FIB-4>2.67 occurring in 816 individuals. Linking these data with FibroScan referral and appointment attendance is ongoing and will continue during the intervention phase, which is expected to commence on February 1, 2024.

**Conclusions:**

We will determine the feasibility and effectiveness of automated APRI and FIB-4 reporting on the monthly rate of transient elastography referrals, liver cirrhosis diagnosis, and linkage to specialist care.

**Trial Registration:**

Australian New Zealand Clinical Trials Registry ACTRN12623000295640; https://tinyurl.com/58dv9ypp

**International Registered Report Identifier (IRRID):**

DERR1-10.2196/56607

## Introduction

### Background

Liver cirrhosis is a major global public health threat; from 1990 to 2017, the global estimate of compensated cirrhosis prevalence increased from 66 million cases to 112 million cases [1], and liver cirrhosis directly causes millions of deaths each year due to liver failure and liver cancer [2,3]. Without timely treatment of chronic liver disease of any cause, individuals are at risk of developing liver cirrhosis [4].

People with compensated cirrhosis receive the greatest benefit from risk factor modification and prevention programs to reduce liver decompensation and improve early liver cancer detection [1]. Curative options are available for early-stage hepatocellular carcinoma; however, most cases are diagnosed at an advanced stage and median survival is only 18% [5], highlighting the importance of early diagnosis [6]. Early-stage liver hepatocellular carcinoma can be identified through surveillance with 6 monthly liver ultrasounds in people with cirrhosis. It is, therefore, imperative that novel population-based screening strategies are explored to identify people with cirrhosis early to facilitate timely linkage to specialist care, including liver cancer surveillance programs.

Transient elastography (FibroScan) has revolutionized the diagnosis of cirrhosis for people with liver disease, providing a noninvasive alternative to liver biopsy [7]. However, at a population level, transient elastography is an unsuitable screening method for cirrhosis due to limited access and cost. Blood-based biomarker algorithms that use readily available blood test parameters, such as the Aspartate Transaminse (AST)–to-Platelet Ratio Index (APRI) [8] and Fibrosis-4 (FIB-4) index [9], have, therefore, been developed as an affordable, accessible screening tool for liver cirrhosis that can triage the need for transient elastography [10].

### Prior Work

Studies in the United Kingdom have used specialized software to automate reflex FIB-4 calculation and reporting on frequently requested blood tests in primary care practice, to increase the diagnosis of people with liver disease and link them to specialist care within public-funded National Health Service networks of the United Kingdom [11,12]. While the iLFT (Intelligent Liver Function Testing) program has proven effective, it requires significant resourcing and may be challenging to translate to privately funded primary care models in countries such as Australia. In contrast, automated reporting of liver fibrosis indices such as APRI and FIB-4 on routinely ordered blood tests by pathology providers does not require specialized software or resourcing and facilitates broad accessibility of results to patients and health workers.

### Study Goal

In this study (CAPRISE [Cirrhosis Automated APRI and FIB-4 Screening Evaluation]), we will determine the feasibility and effectiveness of automated APRI and FIB-4 reporting and provision of brief liver cirrhosis information on the monthly rate and proportion of transient elastography referrals, liver cirrhosis diagnosis, and linkage to specialist hepatology care. We have partnered with a large pathology service in Victoria to pilot a population-level liver cirrhosis screening package, which comprises automated calculation and reporting of APRI and FIB-4 on routinely ordered blood tests and provision of brief information about liver cirrhosis and a web link for transient elastography referral.

### Hypothesis

Automated calculation and reporting of APRI and FIB-4 on routinely ordered blood tests and provision of information about liver cirrhosis, including a link for transient elastography referral, will increase the diagnosis of liver cirrhosis and linkage to specialist care.

### Objectives

The primary objectives of this study are given below.

First, this study aims to measure the monthly rate and the number (proportion) of individuals with APRI greater than 1.0 and FIB-4 greater than 2.67 among people who undergo automated APRI and FIB-4 calculation on blood test events performed through a single Australian pathology service.

Second, this study aims to measure the monthly rate of transient elastography referrals received after the introduction of automated APRI and FIB-4 reporting, compared with the preintervention period.

Third, this study aims to measure the monthly rate and the number (proportion) of transient elastography tests performed in those with an elevated APRI and or FIB-4 score during the intervention period, compared with the preintervention period.

Fourth, this study aims to measure the monthly rate and the number (proportion) of individuals with APRI greater than 1.0 or FIB-4 greater than 2.67 that have cirrhosis range median stiffness (kPa) confirmed on FibroScan.

Finally, this study aims to measure the number (proportion) of individuals with cirrhosis range FibroScan during the intervention period that is linked to specialist care, compared with the preintervention period.

The secondary objective of this study is to describe the sociodemographic factors associated with (1) APRI greater than 1.0 and FIB-4 greater than 2.67, (2) attendance at FibroScan appointment, (3) cirrhosis diagnosed on FibroScan, and (4) linkage to specialist care.

## Methods

### Ethical Considerations

A waiver of consent was granted for this study by the institutional ethics committee (St Vincent’s Hospital Melbourne human research ethics committee project ID 81922, Local Reference LRR 315/21).

### Study Methodology

#### Study Design

A pilot, prospective, single-arm, intervention approach was followed for this study.

#### Study Setting

This study was conducted at the FibroScan clinic of a single tertiary center linked to a large pathology service, which provides both public and privately funded pathology services for the general population in regional and urban areas of Victoria, Australia.

### Primary Outcome Measurement

The primary outcomes prospectively commenced on August 1, 2023, for 6 months before intervention and will continue until the conclusion of the intervention period on July 31, 2024. The impact of the intervention will be determined by comparing primary outcome measures during the intervention period to the 6 months prior to the intervention (standard of care).

### Definitions

#### Comparator Group (Standard of Care)

Currently, APRI and FIB-4 scores are not routinely reported anywhere in Australia. Prior to the intervention, blood tests will be reported per standard of care. APRI and FIB-4 scores will be calculated automatically by the pathology service from the start of the study but will not be reported to ordering clinicians until the intervention period. FibroScans are requested through a paper-based referral form by general practitioners or specialists, which is then faxed to the hospital for triage and booking.

#### Intervention (In Addition to Standard of Care)

St Vincent’s Pathology automatically calculates and reports APRI and FIB-4 scores if platelet count, alanine transferase (ALT) level, and AST level are requested together in a single blood test by primary care physicians. This is provided free of charge. The result will be reported to the requesting primary care physician (both in paper format and online), along with a brief interpretation of the APRI and FIB-4 results, their relevance to liver cirrhosis screening, and a web link for further web-based information. No additional testing or appointments are scheduled as part of this study in addition to the standard of care.

Two separate pathways will be used by St Vincent’s Pathology to notify clinicians of our new APRI and FIB-4 reporting format approximately 1 month prior to the commencement of the intervention period. (1) The St Vincent’s Pathology standard liver function test result report will include a comment stating that APRI and FIB-4 scores will be reported as of the date of project commencement. (2) There will be a new banner included on the St Vincent’s Pathology website notifying clinicians of the availability of automatic reporting of APRI and FIB-4 scores on blood test events and the date that this reporting will commence. This will have a web link to further information on the APRI and FIB-4 score, as well as information about chronic liver disease.

The additional information on the website includes (1) how APRI and FIB-4 are calculated and how it is used, (2) how to interpret the APRI and FIB-4 result, (3) what further tests would be recommended if someone has an abnormal APRI result, (4) further general information about chronic liver disease and liver cirrhosis, and (5) a link to a web-based referral form for the St Vincent’s Hospital FibroScan service to book a confirmatory FibroScan to determine whether the patient has liver cirrhosis.

### Recruitment

All individuals aged 18 years and older who have liver function tests and full blood count requested by a community-based health worker and performed at St Vincent’s Pathology between August 1, 2023, and July 31, 2024, will be recruited. There will be no active recruitment of participants for this study.

This study is an evaluation of a health system–wide introduction of routine reporting of APRI calculation, information on liver cirrhosis, and a web link through St Vincent’s Pathology to improve the quality of care for people with liver disease; therefore, no individuals will individually consent to participate. No personally identifying information will be collected and all data supplied to the research team will be deidentified by St Vincent’s Pathology and transient elastography booking and reporting teams, respectively. Variables including 10-year age category, sex, and postcode will be collected as part of the study but will not allow identification of individuals, as they are broad categories expected to contain more than 1000 persons per strata per month during the study period.

The exclusion criteria of this study include children aged 18 years or younger and blood tests performed during a hospital inpatient stay, or requested by hospital outpatient service, defined by St Vincent’s Pathology records (blood collection site).

### Study Timeline

#### Preintervention Period

Between August 1, 2023, and January 31, 2024, St Vincent’s Pathology will prospectively collect data on all ALT, AST, and platelet count test results conducted, and APRI and FIB-4 scores will be automatically calculated by the St Vincent’s Pathology data management system, but not reported to the ordering clinician. These data will be prospectively collected from St Vincent’s Pathology by a member of the study team, along with exposure variables outlined above. During the same time period, outcome measures per month will be prospectively collected from the St Vincent’s hospital electronic medical records by a member of the research team (number of FibroScan referrals, number [proportion] of scheduled FibroScan appointments attended, number [proportion] diagnosed with cirrhosis, and number [proportion] linked to specialist care).

#### Intervention Period

From February 1, 2024, to July 31, 2024, St Vincent’s Pathology will commence inclusion of automatic calculation of APRI and FIB-4 score reporting on all pathology blood testing events where an AST, ALT, and platelet count are ordered. St Vincent’s Pathology will provide for these testing events: (1) the APRI result, (2) the FIB-4 result, (3) an explanation of what APRI and FIB-4 measure and what cutoffs are used to define cirrhosis, and (4) recommendations for further investigation of elevated APRI or FIB-4 with transient elastography (FibroScan) and performance of a liver disease screen if underlying liver disease is not known. In addition, a web link to the St Vincent’s Pathology website for liver disease and cirrhosis information page will be included on all blood test reports where APRI and FIB-4 are calculated, with a provided URL link to a unique St Vincent’s Hospital FibroScan referral form. This link will be distinct from the standard fax-based St Vincent’s FibroScan referral form to allow measurement of referrals specifically generated in response to St Vincent’s Pathology APRI or FIB-4 score calculations during the study period.

### Statistical Analysis Plan

#### Outcome Measures

Data will be analyzed per test event and per individual tested.

Primary outcomes of this study include the number of APRI and FIB-4 scores calculated on blood test events conducted during the study period, overall and per month; the number (proportion) of APRI scores that are greater than 1.0 and FIB-4 scores that are greater than 2.67; number of FibroScan referrals overall and per month, operationalized as number of completed FibroScan referrals generated online using the recommended St Vincent’s Pathology FibroScan referral link on the request slip, or St Vincent’s Pathology APRI or FIB-4 calculation web page referral form link; number of individuals with an APRI greater than 1.0 or FIB-4 score greater than 2.67 who undergo a FibroScan test at St Vincent’s Hospital Melbourne overall and per month; proportion of individuals with an APRI greater than 1.0 or FIB-4 score greater than 2.67 who undergo a FibroScan test at St Vincent’s Hospital Melbourne; the number of individuals per month who undergo FibroScan at St Vincent’s Hospital Melbourne who have cirrhosis diagnosed (liver stiffness measurement >12.5kPa); the number (proportion) of individuals with an APRI greater than 1.0 or FIB-4 score greater than 2.67 who have cirrhosis confirmed by FibroScan; the number (proportion) of individuals with etiology-specific cirrhosis range FibroScan results that are referred to the specialist liver clinic; and the number (proportion) of individuals with etiology-specific cirrhosis range FibroScan results that attend to the specialist liver clinic appointment.

The secondary outcomes of this study include the number (proportion) of individuals, stratified by age category, sex, and postcode who have elevated APRI greater than 1.0 and FIB-4 score greater than 2.67, attend the FibroScan clinic for further testing, have etiology-specific cirrhosis range FibroScan scores (kPa), and attend a liver specialist clinic appointment.

#### Exposure Variables Collected

Sex, age category (deciles), socioeconomic status of residence (defined by Australian Bureau of Statistics Socio-Economic Index Areas [SEIS] data matched to postcode), urban versus regional place of residence (defined by SEIS data matched to postcode), and liver disease indication recorded on FibroScan requests will be collected.

### Analysis Methods

The number of APRI and FIB-4 scores generated and the proportion of APRI and FIB-4 scores in the cirrhosis range will be described overall and rates per month will be calculated.

The number (proportion) of individuals with an elevated APRI or FIB-4 who are referred for a FibroScan will be described overall and per month; and the number of FibroScan referrals after the intervention will be compared with the number of FibroScan referrals prior to the intervention by logistic regression modeling (time series analysis with the timepoint of interruption defined as February 1, 2024, when the reported APRI score intervention was introduced).

Similarly, the number (proportion) of individuals with an elevated APRI or FIB-4 who are referred for a FibroScan and who attend their FibroScan appointment, overall and per month, will be described and compared with the number (proportion) of FibroScan appointments attended prior to intervention using linear regression modeling, as well as the number (proportion) of individuals with FibroScan results in cirrhosis range that are referred and attend specialist liver clinic appointments.

The number and proportion of individuals with an elevated APRI or FIB-4 who had cirrhosis confirmed by FibroScan will be described, and sensitivity, specificity, and positive and negative predictive values of APRI for cirrhosis determined by confusion matrix. The area under the receiver operating characteristic curve of APRI and FIB-4 for cirrhosis diagnosis will also be calculated by receiver operating characteristic analysis.

The bivariate distribution of sociodemographic (sex, age category, and urban or regional place of residence [defined by postcode using SEIS data]) and clinical variables (liver disease indication) within the proportion of people with elevated APRI and FIB-4 score, who attend FibroScan testing, have elevated FibroScan results, and attend the liver specialist clinic will be determined and compared with their counterparts using chi-square test for categorical data, 2-tailed Student *t* test for parametric continuous data, and Wilcoxon rank-sum test for nonparametric continuous data as appropriate.

A 2-tailed *P* value of .05 will be used as the significance threshold for all analyses. All analyses will be performed using STATA (version 17; Stata Corporation).

### Sample Size Justification

We have conducted a preliminary analysis of the number of AST and platelet count testing events that were performed at St Vincent’s Pathology over a 12-month period (2019). These data showed approximately 11,000 people known to St Vincent’s hospital clinics, and a further 4400 people in the community that had AST and platelet count testing events, that would have allowed APRI calculation to be performed, proving the feasibility of reaching large numbers within the community for cirrhosis screening.

For the comparison of rate of referral for FibroScan before and after the intervention, we estimate that we will be powered to detect a 10% difference in rates of FibroScan referral before and after the intervention. For the logistic regression analyses to determine the difference in proportion of APRI scores leading to referral before and after providing cirrhosis information and FibroScan referral link information (overall and by subgroup), and for determining the proportion of FibroScan referrals that are attended (overall and by subgroup), we are powered to detect a 5% or greater difference between the intervention and historical control groups with power 90% and a sample size of 1100 per subgroup. The above numbers of more than 15,400 individuals having APRI calculations within 12 months suggest that we will be adequately powered to explore our end points including stratified by sex, urban versus rural place of residence, age category, socioeconomic quartile of place of residence, and indication for FibroScan (all of which are expected to have >1000 individuals in each subgroup).

## Results

**Figure 1:**
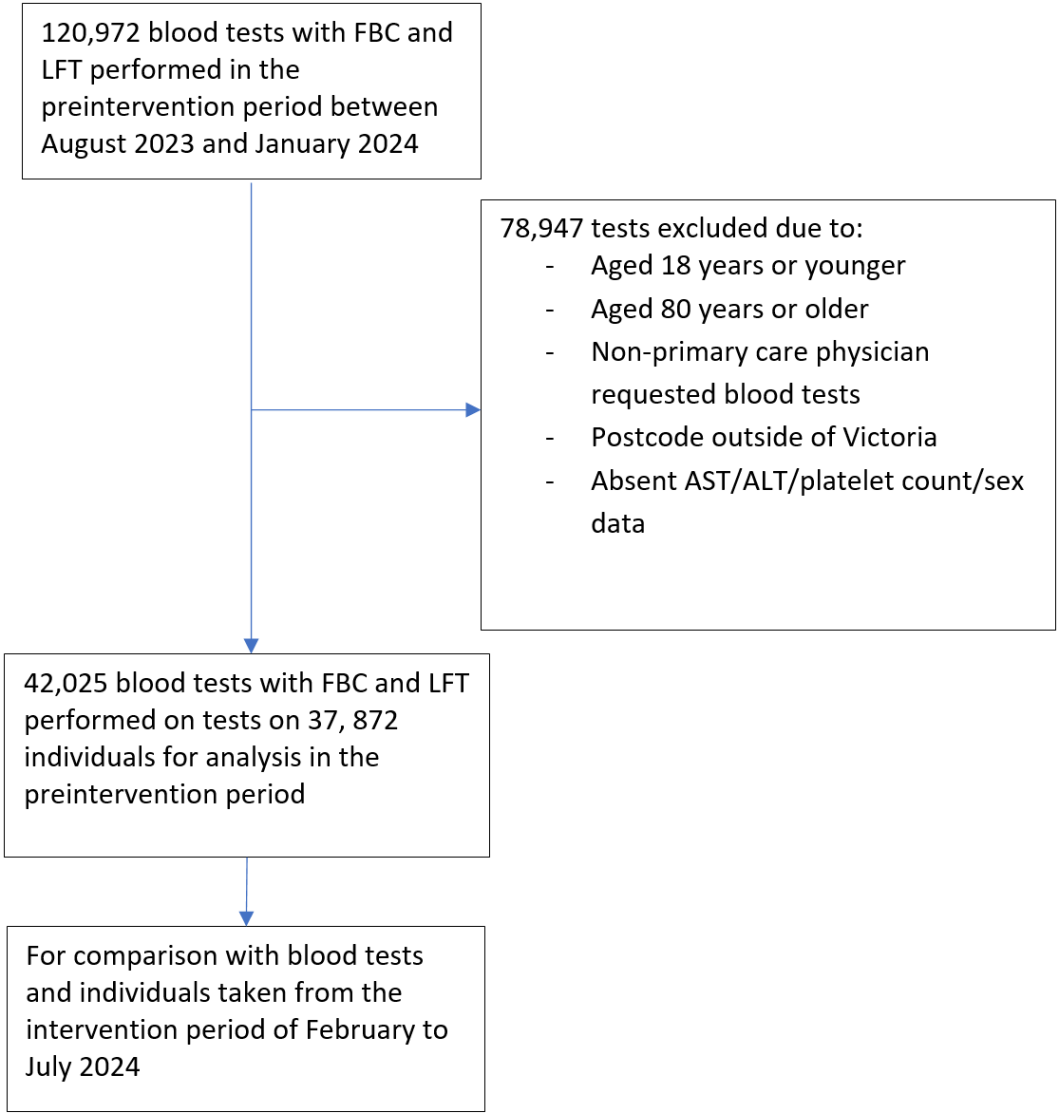
Flow diagram of current data from the preintervention period of blood tests performed at St Vincent’s Pathology and included in the interim analysis. ALT: alanine transferase; AST: aspartate transferase; FBC: full blood count; LFT: liver function test.

As of January 2024, in the preintervention phase of this study, a total of 120,972 tests were performed by the laboratory. Of these tests, 78,947 (65.3%) tests were excluded, with the remaining 42,025 (34.7%) tests on 37,872 individuals meeting inclusion criteria with APRI and FIB-4 being able to be calculated (Figure 1). Of these, 1.3% (n=531) had elevated APRI greater than 1 occurring in 446 individuals, and 2.3% (n=985) had elevated FIB-4 greater than 2.67 occurring in 816 individuals (Table 1). Linking these data with FibroScan referral and appointment attendance is ongoing and will continue during the intervention phase.

Data analysis is expected to begin following 6 months of intervention in August 2024, and the publication of results will then follow.

**Table 1:** Proportion of blood tests and individuals with elevated Aspartate Transaminse–to-Platelet Ratio Index (APRI )and Fibrosis-4 (FIB-4) values.

	Total test events (n=42,025), n (%)	Individuals (n=37,872), n (%)
Elevated ALT^a^	16,760 (39.9)	15,285 (40.4)
Elevated APRI>1	531 (1.3)	446 (1.2)
Elevated FIB-4>2.67	985 (2.3)	816 (2.2)

^a^ALT: alanine transferase.

## Discussion

### Scientific Contribution

The results from this study will contribute to knowledge gaps surrounding the prevalence of individuals with compensated cirrhosis in a community setting and the use of services instigated through primary care.

### Strengths and Limitations

This study will determine the feasibility, effectiveness, and implementation requirements of a population-level screening program for liver cirrhosis in Australia.

Strengths of this study include its prospective study design and large population-based cohort, which is representative of the Australian population.

Automated liver fibrosis index calculation and reporting by pathology services using frequently requested blood tests is a low-cost, potentially scalable approach to population-level liver cirrhosis screening and may also raise awareness of chronic liver disease among health workers and the community.

Study limitations include the limited set of clinical and demographic variables available from routinely collected pathology data to inform risk factors for cirrhosis on a population scale.

Resource requirements and logistics of specialist referral may limit the scalability of blood test–based cirrhosis screening on a population level.

### Conclusions

In summary, we aim to test the feasibility of determining the prevalence of elevated noninvasive tests, APRI, and FIB-4 and the use of services that would aid in the diagnosis of liver cirrhosis and linkage to specialist care.

## Abbreviations

ALT: alanine transferase
APRI: Aspartate Transaminse–to-Platelet Ratio Index
AST: aspartate transferase
CAPRISE: Cirrhosis Automated Aspartate Transaminse–to-Platelet Ratio Index and Fibrosis-4 Screening Evaluation
FIB-4: Fibrosis-4
iLFT: Intelligent Liver Function Testing
SEIS: Socio-Economic Index Areas

## Data Availability

The data sets generated or analyzed during this study are available from the corresponding author on reasonable request.
